# Massive application of the SARS-CoV-2 diagnostic test: simulation of its effect on the evolution of the epidemic in Spain

**DOI:** 10.1017/S0950268820002289

**Published:** 2020-09-29

**Authors:** Jacobo López-Abente, Clara Valor-Suarez, Gonzalo López-Abente

**Affiliations:** 1 Department of Biochemistry and Molecular Biology, Chemistry School, Complutense University of Madrid, Madrid, Spain; 2 Department of Ophthalmology, Rey Juan Carlos Hospital, Madrid, Spain; 3 Former Researcher of National Center for Epidemiology, Madrid, Spain

**Keywords:** COVID-19, epidemic, simulations, individual contact models

## Abstract

In Spain, the epidemic curve caused by COVID-19 has reached its peak in the last days of March. The implementation of the blockade derived from the declaration of the state of alarm on 14th March has raised a discussion on how and when to deal with the unblocking. In this paper, we intend to add information that may help by using epidemic simulation techniques with stochastic individual contact models and several extensions.

In Spain, the epidemic curve caused by COVID-19 has reached its peak in the last days of March. The implementation of the blockade derived from the declaration of the state of alarm on 14th March has raised a discussion on how and when to deal with the unblocking. The main points of this discussion are the containment of the epidemic and the protection of the economy that is seriously threatened. The opinion of technicians and public health experts is that the lockdown must be extended to avoid a rebound of the epidemic, a second wave that could be even more serious than the first. This could happen if containment is lifted too early [1].

In this paper, we intend to add information that may help to make the right epidemic intervention decisions regarding public health. Three possible scenarios are shown and the temporal evolution of various health/disease indicators is simulated using mathematical models. The simulated scenarios were: (1) non-intervention, (2) temporary locking/confinement and (3) temporary locking/confinement plus mass determination of infectious status with self-isolation in the event of being infective.

The dynamics of infectious processes are studied by mathematical models that include deterministic compartmental models, stochastic individual-contact models and stochastic network models [2]. The first two evaluate the epidemiological situation using a theoretical formulation of population distribution in different categories called compartments. That is why SIR models (susceptible (S), Infected (I), Recovered (R)) were initially selected. These models can be adapted to different pathologies through extensions that involve including new compartments.

In our study, the epidemic has been simulated by stochastic individual contact models using the EpiModel open access package running under the R statistical environment. This model adds four more compartments to SIR: asymptomatic infected (E), hospitalisation (H), quarantine (Q) and deceased (F) (‘SEIQHRF’) [3, 4]. The model and extensions have been evaluated using COVOID (COVID-19 Open-Source Infection Dynamics) [5]. These models explicitly represent individual units in the population and the contacts between them as unique, discrete events.

Simulations have been performed on a theoretical population of 100 000 people followed for 2 years (days used as unit of time). Given the stochastic component of a part of the procedure, eight chains (repeated simulations) have been generated which have been averaged to obtain the final result. Our outbreak simulation has started with six cases and the basic reproductive number was 1.9 for the first 10 days. Simulations require specifying initial parameters associated to the population (population size, number of initial cases, general and hospitalised mortality rates, fatality rates in the infected, hospitalisation capacity in ICU, etc.) and others related to the simulation itself as commented below. The data, procedures and software that support the findings of this study are openly available in [3], including initial model parameters.

In Spain, the first cases diagnosed and admitted to hospitals occurred in Madrid on the last days of February and the alarm status was established on 14th March (23 days after epidemic onset). The reactivation of some industrial sectors started on 13th April. During the summer holidays (July and August) a gradual activity increase and subsequent decrease in social precautions is expected. To simulate this sequence of social phenomena we have resorted to the ‘act.rate.i’ parameter that corresponds to the number of exposure events (acts) between infectious and susceptible individuals per day, therefore indirectly represents the ‘social distance’. Such parameter is shown in Figure 1a, where the ordinates expresses the ‘social distance’ and the abscissa are the days since the beginning of the epidemic. The simulation shows the consequences of variations in act.rate.i on the population (compartments). To this end, a gradual increase in act.rate.i has been defined after the removal of the blockade followed by stabilisation on day 190.

**Fig. 1.**
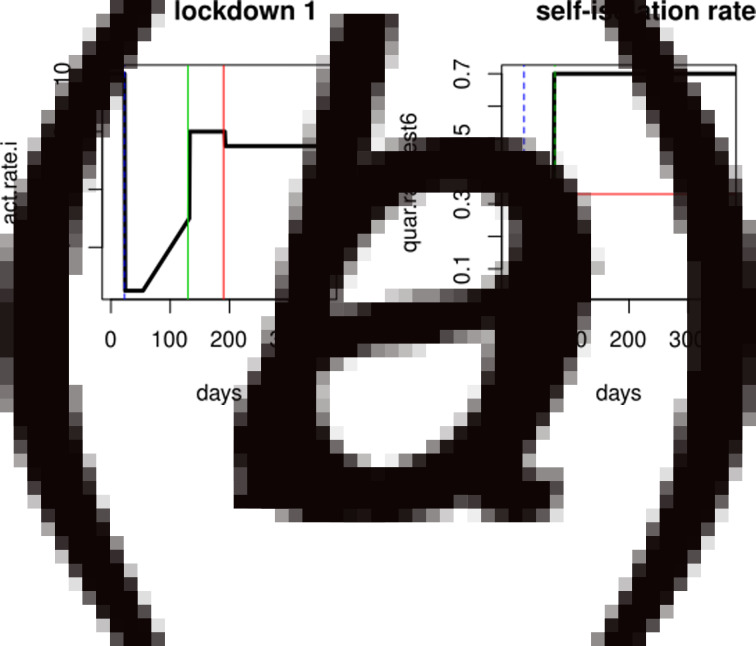
Simulation parameters. (a) Evolution of the activity rate (bold line) in ‘Lockdown 1’, vertical green and red lines represent summer months (holidays). (b) Evolution of self-isolation rate (bold line). Red line: quarantine rate in ‘Lockdown 1’ (no PCR testing); blue line: alarm declaration; green line: beginning of the massive PCR testing.

To simulate the impact that polymerase chain reaction (PCR) testing in symptomatic individuals has over the evolution of the epidemic, the ‘daily quarantine’ rate (‘quar.rate.test’) that varies over time has been added to ‘Lockdown 1’ (Fig. 1b). According to this daily rate, symptomatic infected individuals (I, PCR positive) will enter self-isolation (Q). Asymptomatic infected people cannot enter self-isolation because they do not yet know they are infected. The default start value (1/30) is a low rate that reflects low community awareness or noncompliance with self-isolation requirements or practices.

The self-isolation rate evolution shown in Figure 1b has been constructed as follows: 1/30 during the first 23 days (alarm declaration), gradual increase until 1/3 on the following 30 days, 1/3 maintained for 20 days (massive testing starts) and 0.7 the rest of the year due to testing intervention. People who know their infectious status are assumed to be self-isolating. Probability of transmitting the infection at each exposure event (‘inf.prob.q’) for interactions between infectious people in quarantine (Q) and susceptible individuals (S) in both simulations was 0.015. Protective measures are assumed to be maintained as long as the virus continues to circulate. The baseline situation (without any intervention) has also been simulated for comparative purposes. In Figures 2 and 3 we show the simulation result (consequences of the two scenarios): (1) ‘Lockdown 1’ and (2) massive application of the PCR test respectively. The evolution of the compartments over a year is represented. ‘Lockdown 1’ scenario (Fig. 2) shows a strong infection rebound after 200 days (September) of evolution of the epidemic. However, if the intervention based on the PCR test is massively applied to the population, the second wave of infection is practically avoided since the asymptomatic infectious will also detected and isolated (Fig. 3).

**Fig. 2.**
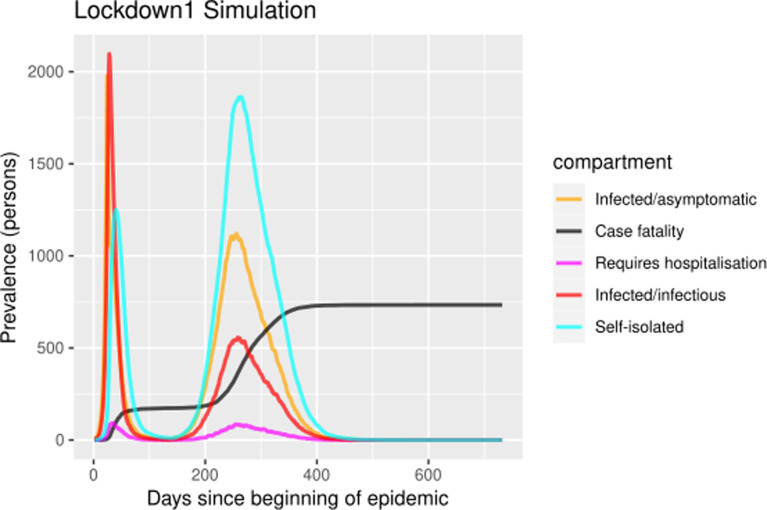
Simulation of prevalence numbers for each compartment in ‘Lockdown 1’ with gradual incorporation to activity.

**Fig. 3.**
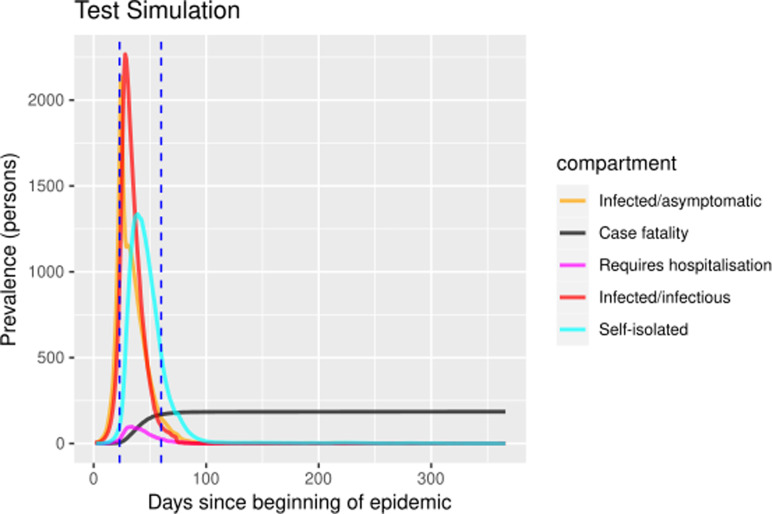
Prevalence numbers for each compartment in simulation of ‘Lockdown 1’ including the massive SARS-CoV-2 laboratory test. Vertical blue lines at day 23 and day 60 represent the alarm state and the massive laboratory test respectively. The simulations spanned 700 days. The figure only shows the first year because there were no subsequent events.

Table 1 shows quantitative data for the seven compartments (viz., susceptible, infected-asymptomatic, infected, self-isolated, hospitalised, recovered and deaths), to assess the magnitude of the consequences of the epidemic. Data obtained from the three scenarios: (1) baseline situation without any intervention, (2) ‘Lockdown 1’ and (3) massive application of the laboratory test. Three time points are shown: 50, 200 and 300 days. Without any intervention, 95% of the population would become infected, 2% dying in less than 3 months. In ‘Lockdown 1’ there is an important rebound that multiplies by 2 the impact of the first wave. If we apply these percentages to a population of 6 million people (Madrid e.g.), the model predicts 10 000 deceased in the first wave and 20 000 in the second wave. Finally, the intervention on the quarantine rate facilitated by the massive execution of the diagnostic test would avoid this second wave. This second wave has started in Madrid and other regions of Spain in the early days of September.

**Table 1.** Numerical results of the simulations

		Basal			Lockdown 1			Test		
Days	Compartment	Count	%	Population^a^ 6 million	Count	%	Population 6 million	Count	%	Population 6 million
50	Susceptible	4150	4.15	48 970	90 608	90.61	5 436 465	89 949	89.95	5 396 918
50	Infect/asympt	3197	3.2	191 828	361	0.36	21 653	383	0.38	22 973
50	Infected	20 796	20.8	1 247 783	334	0.33	20 063	355	0.35	21 293
50	Self-isolated	5539	5.54	332 333	960	0.96	57 570	1016	1.02	60 975
50	Hospitalised	986	0.99	59 183	53	0.05	3165	51	0.05	3060
50	Recovered	63 923	63.92	3 835 380	7593	7.59	455 603	8148	8.15	488 880
**50**	**Case fatality**	**1459**	**1.46**	**87** **525**	**140**	**0.14**	**8370**	**148**	**0.15**	**8850**

200	Susceptible	2482	2.48	148 928	87 868	87.87	5 272 103	89 041	89.04	5 342 445
200	Infect/asympt	0	0	0	283	0.28	16 980	0	0	15
200	Infected	0	0	0	126	0.13	7553	0	0	8
200	Self-isolated	1	0	60	353	0.35	21 158	2	0	143
200	Hospitalised	0	0	0	13	0.01	803	0	0	8
200	Recovered	95 693	95.69	5 741 565	11 360	11.36	681 578	10 963	10.96	657 780
**200**	**Case fatality**	**2033**	**2.03**	**121** **980**	**186**	**0.19**	**11** **160**	**185**	**0.18**	**11** **070**

300	Susceptible	2757	2.76	165 405	63 209	63.21	3 792 563	89 137	89.14	5 348 220
300	Infect/asympt	0	0	0	674	0.67	40 448	1	0	30
300	Infected	0	0	0	357	0.36	21 443	0	0	0
300	Self-isolated	0	0	15	1272	1.27	76 290	1	0	68
300	Hospitalised	0	0	0	55	0.06	3323	0	0	0
300	Recovered	95 515	95.52	5 730 923	34 152	34.15	2 049 098	10 964	10.96	657 818
**300**	**Case fatality**	**2033**	**2.03**	**121** **995**	**565**	**0.57**	**33** **908**	**185**	**0.18**	**11** **093**

Quantitative data for the seven compartments (viz., susceptible, infected-asymptomatic, infected, self-isolated, hospitalised, recovered and deaths). Prevalence of people in each compartment at day 50, 200 and 300 after epidemic onset for three scenarios: (1) baseline situation (‘Basal’) without any intervention, (2) temporary locking/confinement (‘Lockdown 1’) and (3) temporary locking/confinement plus the massive application of the laboratory test (‘Test’).

a Estimated events in a simulated 6 million population.

The simulation poses a theoretical situation that may or may not be partially fulfilled and has some limitations [6]. In the first place, the parameters shown in the PCR test scenario are most probably somewhat excessively optimistic, but this reduction in the second epidemic wave can also be observed in other worse scenarios (not shown). However, the recent publication of the possibility of doing a self-administered, low-cost test to detect the virus in saliva samples [7] could provide added value to these simulations. Setting a self-isolation rate of 0.7 assumes rigorous self-isolation. However, the construction of the vector of daily values that it represents cannot be validated yet. In most scenarios, highly effective contact tracing and case isolation is enough to control a new outbreak of COVID-19 within 3 months [8]. However, in the current situation, the pandemic requires extraordinary measures since the virus has spread throughout the country very quickly and is causing very serious clinical symptoms in people with underlying problems such as chronic obstructive pulmonary disease, diabetes or hypertension, which are highly prevalent in the elderly population [9].

In addition, there are historical experiences were second waves can be more threatening than the first ones, and that surely exhausted the susceptible population. Another limitation is that the model does not include critical aspects such as differential risk by age group. It is certain that an ageing population will suffer worse consequences. It also does not contemplate the arrival of infected people (imported cases).

It is important to highlight the relevance of simulating broad periods of time which have helped to analyse the complete evolution of the epidemic, including the second wave. Another aspect that should be taken into account is the size of the simulated population. This should be large enough not only to emulate the current situation of big cities where the epidemic is having greater impact, but also because simulations with a small population do not show the potential impact of the epidemic. However, such large-scale simulations are time consuming and computer-power demanding.

This simulation provides a more hopeful scenario on the evolution of the epidemic, which by day 50 of its beginning, has already registered 177 000 cases and 18 500 deaths in Spain. The simulation results support the validated ‘trace, test and treat’ strategy for the epidemic experience in the Republic of Korea [10]. In this country, for a total population of 56 million people, 94 000 PCR tests were performed to confirm suspected cases of COVID-19. The singular difference between the Republic of Korea and other countries is the quick intervention that made the blockade unnecessary. To conclude, we must comment that these notes, due to all their limitations, should be considered an academic exercise that points towards two conclusions: first, the potential danger of a bigger second epidemic wave and second, the detection of possible transmitters of the infection through massive PCR testing may help to avoid such potential situation.

## Data Availability

The data and software that support the simulation results of this study are openly available in [3].
